# Comparative transcriptome and metabolome profiles of the leaf and fruits of a Xianjinfeng litchi budding mutant and its mother plant

**DOI:** 10.3389/fgene.2024.1360138

**Published:** 2024-02-23

**Authors:** Ning Xu, Xian-quan Qin, Dong-bo Li, Yan-jie Hou, Chen Fang, Shu-wei Zhang, Jing-yi You, Hong-Li Li, Hong-ye Qiu

**Affiliations:** Horticultural Research Institute, Guangxi Academy of Agricultural Sciences, Nanning, Guangxi, China

**Keywords:** amino acids and derivatives, carbohydrates and derivatives, flavonoid metabolome in fruits, leaf folding, litchi fruit

## Abstract

**Background:** Litchi (*Litchi chinensis*) is an important sub-tropical fruit in the horticulture market in China. Breeding for improved fruit characteristics is needed for satisfying consumer demands. Budding is a sustainable method for its propagation. During our ongoing breeding program, we observed a litchi mutant with flat leaves and sharp fruit peel cracking in comparison to the curled leaves and blunt fruit peel cracking fruits of the mother plant.

**Methods:** To understand the possible molecular pathways involved, we performed a combined metabolome and transcriptome analysis.

**Results:** We identified 1,060 metabolites in litchi leaves and fruits, of which 106 and 101 were differentially accumulated between the leaves and fruits, respectively. The mutant leaves were richer in carbohydrates, nucleotides, and phenolic acids, while the mother plant was rich in most of the amino acids and derivatives, flavonoids, lipids and organic acids and derivatives, and vitamins. Contrastingly, mutant fruits had higher levels of amino acids and derivatives, carbohydrates and derivatives, and organic acids and derivatives. However, the mother plant’s fruits contained higher levels of flavonoids, scopoletin, amines, some amino acids and derivatives, benzamidine, carbohydrates and derivatives, and some organic acids and derivatives. The number of differentially expressed genes was consistent with the metabolome profiles. Gene Ontology and Kyoto Encyclopedia of Genes and Genomes pathway-enriched gene expressions showed consistent profiles as of metabolome analysis.

**Conclusion:** These results provide the groundwork for breeding litchi for fruit and leaf traits that are useful for its taste and yield.

## 1 Introduction

Litchi (*Litchi chinensis*) is native to China and has a history of cultivation dating back to 2000 BC (Kilari and Putta, 2016). As a sub-tropical fruit, it is grown in southern China. It is also grown in Australia, Hawaii, India, Madagascar, Mauritius, Myanmar, northern Vietnam, Pakistan, South Africa, and Thailand (Mitra and Pan, 2019). Fresh litchi exports from China accounted for 63.63 million USD in 2021, an increase from 53.2 million USD in the previous year. Guangdong province is the major producer of litchi in China. With the increasing demand for litchi in developing countries, the current market revenue of USD 6.73 billion is expected to increase by 5.5% to reach USD 8.79 billion in the next 5 years. (https://www.mordorintelligence.com/; accessed on 28 September 2023). However, this increased demand can only be met by working to improve the current varieties by increasing their yield, fruit esthetics, and stress tolerance (Yang et al., 2022). In this regard, this exotic fruit is being grown in a wide range of environmental conditions (Sarin et al., 2009). However, introducing it to a wide range of geographical regions is not an easy task and requires continued breeding efforts. Apart from increasing the area under cultivation, breeding for traits like fruit appearance, color, size, and taste can also increase choices for consumers with different taste and health preferences.

Litchi breeding includes selection, budding, artificial hybridization, and mutation breeding. Litchi breeding programs in China have mainly developed new varieties through seed selection, but hybridization has also proved to be a useful method (Sarin et al., 2009). For example, varieties such as “Jumeiren” and “Xiantao” litchi have been developed through hybridization. However, the low budding probability of litchi is a limitation of breeding by this method, and only one variety (Guiping Madonghe litchi) has been reported so far. During the ongoing litchi breeding activities for high-quality fruit and different ripening times, our research group identified a single plant of the “Xianjinfeng” variety that showed significant variation in leaf and fruit phenotypes compared to its parent plant. This variant (hereafter mutant) is a result of budding. This study aims to understand the key metabolomic differences between the leaves and fruits of the mutant and its parent plant. Previous research has shown that the litchi fruit metabolome contains sugars; organic acids; floral volatiles; polyphenols such as flavonoids, catechins, flavonols, and hydroxycinnamoyls (Jiang et al., 2021); amino acids; and other nutrients (Yun et al., 2022). *In vitro* and *in vivo* studies have shown that litchi fruits have anti-obesity, anticancer, antioxidant, anti-atherosclerotic, and neuroprotective activities due to the presence of active metabolites. In addition, consumption of litchi fruit has also shown a wide range of benefits such as hypoglycemic, hepatoprotective, hypolipidemic, hypotensive, and immunomodulatory effects (Zhao et al., 2020). Given these advantages, it is important to understand whether the identified mutant has differences in metabolome and transcriptome profiles. This is important to decide if there are compounds of particular interest and how this mutant can be used effectively in the ongoing breeding program.

Several studies have shown that mutant and wild-type plants can differ in their metabolomes. For example, in a study of *Ginkgo biloba*, the authors found that the leaves had different levels of carotenoids, flavonoids, and other compounds. The study also showed that these changes were associated with different gene expressions in the associated pathways (Wu et al., 2020). Several other studies comparing leaves from the mutant and their parent or wild-type plants have highlighted the presence of differences in photosynthesis, carbohydrate biosynthesis, pigments, compounds of nutraceutical interest, fatty acids, flavonoids, linoleic acid, and organic acids and derivatives (Qiao et al., 2022; Grauso et al., 2023; Han et al., 2023). Although a large number of studies have examined the mechanics of leaf morphogenesis (Guo et al., 2022; Nakata and Takahara, 2022), less has been explored on the metabolome and transcriptome profiles of mutant plants compared with mother plants, especially considering that the mutant leaves show a slightly folded phenotype. However, some studies involving stems indicated that bending is related to stresses, phytohormones, and cell growth mechanisms (Jonsson et al., 2023). In the case of fruits, metabolomics can reveal differences in pigment biosynthesis and accumulation, nutritive compounds, bioactive metabolites, and defense-related metabolites between mutant and wild-type plants. For example, combined metabolomic and transcriptomic analysis revealed the mechanism of color formation in pepper fruits (Liu et al., 2020) and fig (Wang et al., 2017), fruit flavor in Guifei mango (Lv et al., 2022; Wang et al., 2022), and flavonoid/anthocyanin biosynthesis in strawberry (Lin et al., 2022) and jujuba (Shi et al., 2020).

Litchi fruit is directly consumed by humans, and therefore any observable changes in appearance as well as flavor can directly affect farm income and thus the economy associated with it. In this regard, understanding the metabolome and transcriptome differences in the newly identified Xianjinfeng litchi mutant fruits and leaves is a useful strategy. This comparison will not only provide preliminary data on nutritional differences but also potential pathways and genes controlling key metabolites. Therefore, we used ultra-performance liquid chromatography–tandem mass spectrometry (UPLC-MS/MS) to investigate the global metabolites in the leaf and fruit of the mutant and compared them with those of the corresponding organs in the parent plants.

## 2 Materials and methods

### 2.1 Plant material

**FIGURE 1:**
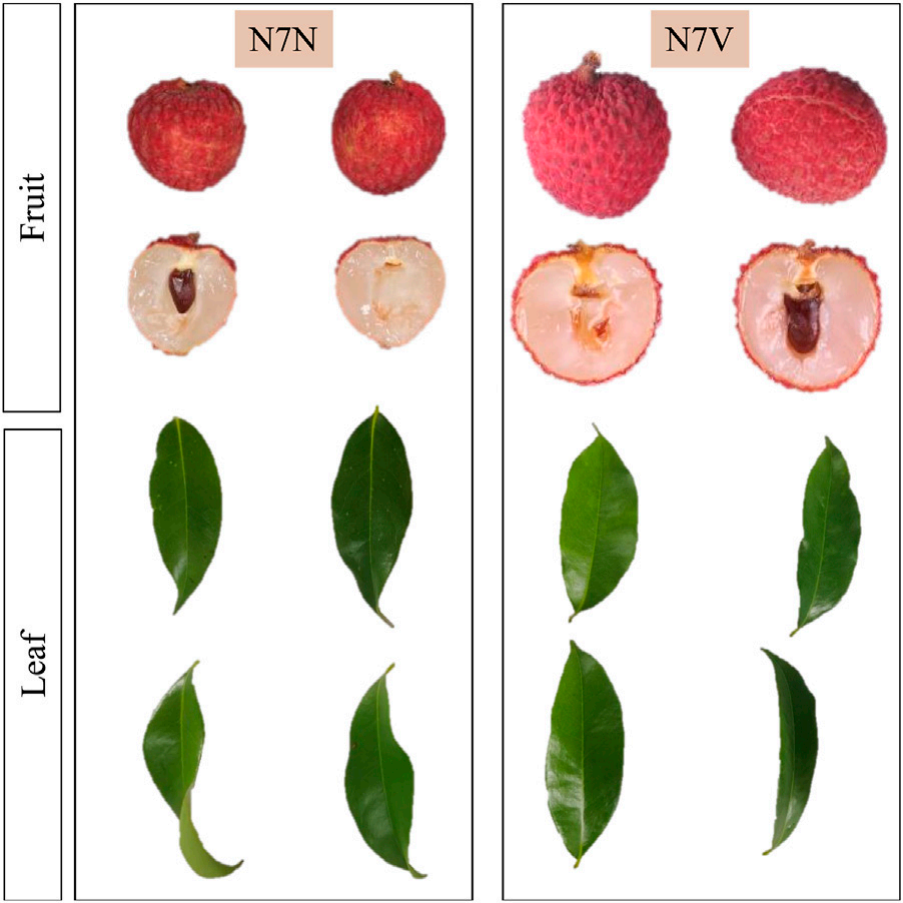
Phenotypic differences in fruits and leaves of the Xianjinfeng mother plant (N7N) and mutant (N7V).

*Litchi chinensis* var. Xianjinfeng single plant buds were grafted onto healthy Heli litchi rootstocks in 2017 in Pingnan District, Guiping City. In 2019, a mutant single plant N7 was found in this batch of grafted seedlings. Two different types of leaves and fruits were found on N7. The leaves N7NL and fruits N7NF of one of them are consistent with the traits of Xianjinfeng. The leaves N7VL and fruits N7VF of the other one have different traits compared to those of Xianjinfeng. The skin of N7VF is turtle cracking, and the peak is raised. The flesh texture is crispier than that of Xianjingeng. The maturity period is 7–10 days less than that of Xianjinfeng. On the contrary, N7NF’s peel exhibits smooth cracking, whereas the mutant plants’ leaves (N7VL) are smooth compared to those of N7NL, which are curled (Figure 1). In 2022, the leaves N7NL and N7VL and fruits N7NF and N7VF of both types of grafts were collected and used for subsequent omics analyses.

### 2.2 Metabolome analysis

Vacuum freeze-dried leaf and fruit samples of N7N and N7V were ground to powder using a grinder (MM 400, Retsch, Haan, Germany). Powder aliquots (100 mg) were extracted at 4°C with 0.6 mL of 70% aqueous methanol. The aliquots were vortexed during the extraction. The extracts were centrifuged (10,000 g) for 10 min and filtered through a microporous membrane (0.22 µm). After aspirating the supernatant, the sample was stored in a sample vial for UPLC-MS/MS analysis. Ultra-performance liquid chromatography (UPLC) (Shim-Pack UFLC SHIMADZU CBM30A, https://www.shimadzu.com.cn/) and tandem mass spectrometry (SHIMADZU Corp., Kyoto, Japan) (MS/MS) (Applied Biosystems 4500 QTRAP) were used for data acquisition. UPLC-MS/MS operating conditions were as previously reported (Yu et al., 2022).

#### 2.2.1 Data analysis

Quality control samples were used for quality control during detection. Principal component analysis (PCA) was calculated based on relative metabolite intensity using the R language (www.r-project.com) and the gmodels (v2.18.1) package. Subsequently, a distance matrix was calculated for all samples, for which hierarchical clustering was used (Chong and Xia, 2018). All metabolites were standardized by z-score, and heat maps were generated using the R package pheatmap (v1.0.12). The Pearson correlation coefficient (PCC) was calculated in R using the cor() function. In addition to PCA, we also performed partial least squares discriminant analysis (PLS-DA) and orthogonal partial least squares discriminant analysis (OPLS-DA) on the metabolomic data (Bylesjö et al., 2006; Worley and Powers, 2013). Differentially accumulated metabolites (DAMs) between different comparison groups were identified using the variable importance in projection (VIP) value from OPLS-DA multivariate statistical analysis and the *t*-test *p* value from univariate statistical analysis on mean (*n* = 3) values of metabolite intensities (Saccenti et al., 2014). Metabolites were considered differentially accumulated if VIP ≥ 1 and *t*-test *p* < 0.05 in the OPLS-DA model. Corresponding compound IDs of DAMs were searched in KEGG. KEGG pathways with significantly enriched DAMs were identified using the hypergeometric test (Kanehisa and Goto, 2000; Kanehisa and Sato, 2020). Metabolite set enrichment analysis was performed to identify the changing patterns of metabolite concentrations in KEGG pathways using the quantitative enrichment analysis mode in the MetaboAnalystR package (Chong and Xia, 2018).

### 2.3 Transcriptome sequencing

The fruit pulp from three fruits were used for total RNA extraction using the Plant Total RNA Purification Kit (made) according to the manufacturer’s instructions. RNA purity testing, quantification, and integrity were performed using a NanoPhotometer (IMPLEN, Los Angeles, CA, United States) and the Qubit RNA Assay Kit in a Qubit 2.0 Fluorometer (Life Technologies, Carlsbad, CA, United States) and the RNA Nano 6000 Assay Kit on the Agilent Bioanalyzer 2100 system (Agilent Technologies, Santa Clara, CA, United States). Sequencing of library preparation and quality control were performed as previously reported (Liu et al., 2019) and sequenced on the Illumina sequencing platform by Gene Denovo Biotechnology Co., Ltd. (Guangzhou, China).

The sequencing data were first processed for quality control using fastp (Chen et al., 2018) to remove reads with adapters, having an N ratio >10%, with all A bases, and those with Q ≤ 20 for more than 50% of the entire read. Moreover, the base composition and quality distribution analyses were performed. The high-quality reads were compared with the reference genome (JAHYJY000000000). According to the comparison results of HISAT2 (Kim et al., 2019), we used StringTie (Pertea et al., 2015) to reconstruct transcripts and used RSEM (Li and Dewey, 2011) to calculate the expression levels of the genes as Fragments Per Kilobase of transcript per Million mapped reads (FPKM) in each sample. The gene expression abundance distribution, PCA, and PCC were computed in R (www.r-project.org). Next, we computed differential gene expression [by using mean FPKM values (*n* = 3)] between N7NL vs N7VL and N7NF vs N7VF using DESeq2 (Love et al., 2014), and differentially expressed genes/transcripts (DEGs/DETs) were screened if the false discovery rate (FDR) was < 0.05 and log2 fold change was > 2. The DEG/DET data were visualized as volcano plots and clustered (hierarchical clustering) in R (www.r-project.org). The DEGs/DETs were mapped to the Gene Ontology (GO) database () and KEGG pathway database (https://www.genome.jp/kegg/pathway.html). A hypergeometric test was applied to find significant GO terms and KEGG pathways to which the DEGs/DETs were significantly enriched.

### 2.4 Quantitative real-time PCR analysis

The RNAs were extracted, quantified, and tested for quality from triplicate samples, as mentioned in Section 2.3. The qRT-PCRs for 13 selected genes were carried out as reported earlier (Li et al., 2013). Primers were designed using Primer Premier 5 (Lalitha, 2000). The qRT-PCR for each reaction mixture was performed using 5 µL 2x qPCR mix, 10 pmol/μL each of forward and reverse primers, 2 µL cDNA, and 2.5 µL ddH_2_O. The reactions were carried out on the QuantStudio™ 5 Real-Time PCR System (Applied Biosystems, Waltham, Massachusetts, United States). The data quality control analysis was performed by using QuantStudio™ Design & Analysis Software (Applied Biosystems, Waltham, Massachusetts, United States). The 2^−ΔΔCT^ method was used for measuring relative gene expression (Livak and Schmittgen, 2001).

## 3 Results

### 3.1 Global metabolome profile of litchi leaf and fruit

**FIGURE 2:**
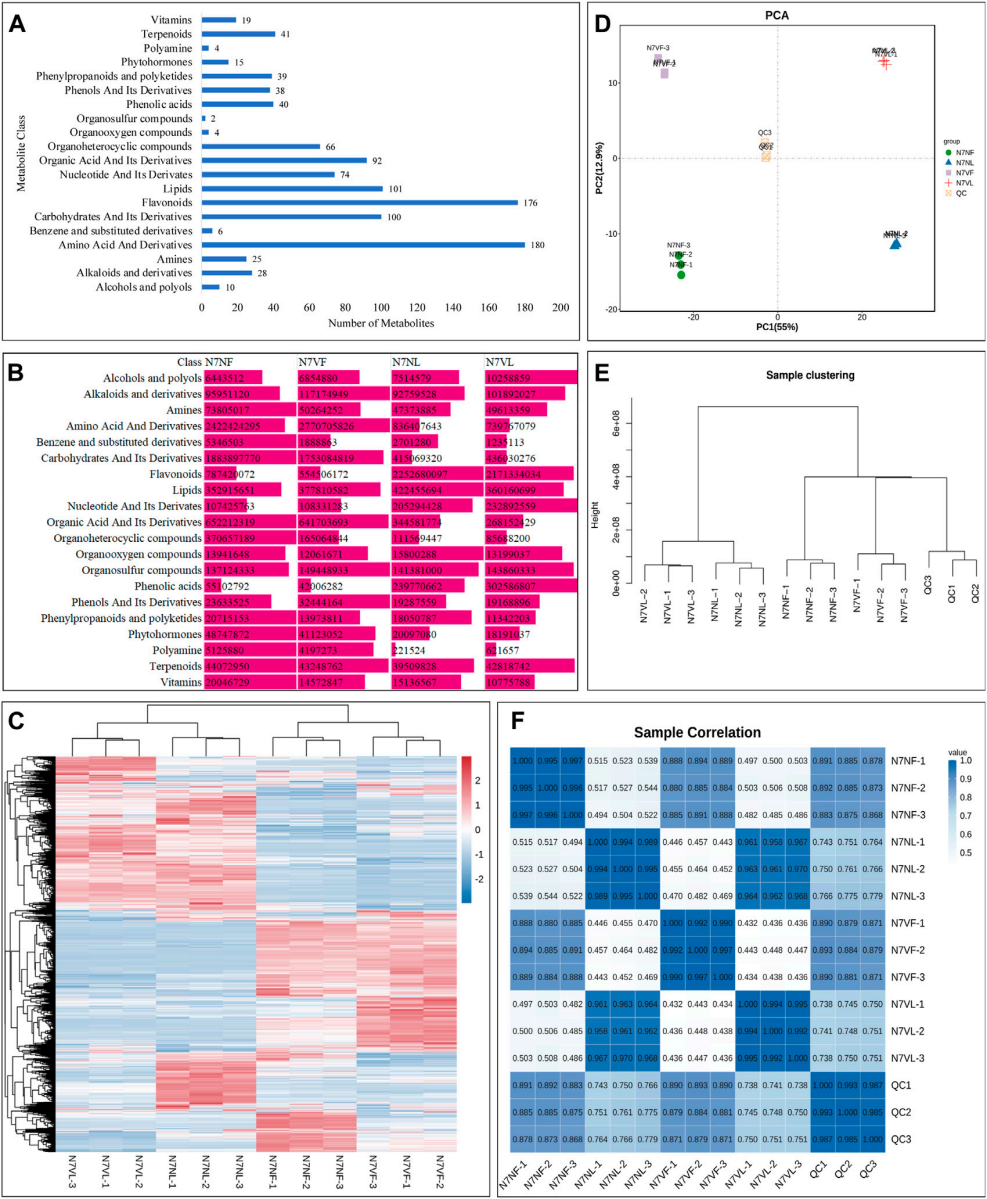
Global metabolite profile of leaves and fruits of N7N and N7V litchi. **(A)** Bar graph showing the number of metabolites detected in each class of compounds. **(B)** Comparison of the sum of intensities of metabolites in each compound class. **(C)** Heatmap of relative intensities of metabolites detected in each replicate of the leaf and fruit of N7N and N7V. **(D)** Principal component analysis, **(E)** hierarchical clustering, and **(F)** Pearson’s correlation analysis based on the relative metabolite intensity in N7N and N7V leaves and fruits. The numbers with sample names represent replicates.

Metabolomic analysis of 12 litchi samples (six fruit and six leaf samples) revealed 1,060 metabolites. These metabolites belonged to 20 compound classes. Amino acids and derivatives constituted the highest number of metabolites (180), followed by flavonoids (176), lipids (101), organic acids and their derivatives (92), and organoheterocyclic compounds (66) (Figure 2A). Leaves had a higher content of alcohols and polyphenols, flavonoids, lipids, nucleotides and derivatives, organooxygen compounds, phenolic acids, and vitamins than fruits, whereas the content of alkaloids and derivatives, amines, amino acids and derivatives, benzene and substituted derivatives, carbohydrates and their derivatives, organic acids and their derivatives, organoheterocyclic compounds, organosulfur compounds, phenols and their derivatives, phenylpropanoids and polyketides, phytohormones, polyamines, and terpenoids was higher in fruits (Figures 2B, C).

In general, N7VL had a higher content of alcohols and polyphenols, alkaloids and derivatives, amines, carbohydrates and derivatives, nucleotides and derivatives, organosulfur compounds, phenolic acids, and terpenoids than N7NL, while the content of the other compounds was higher in N7NL than in N7NV. In fruits, the content of alcohols and polyphenols, alkaloids and derivatives, amino acids and derivatives, lipids, nucleotides and derivatives, organosulfur compounds, phenols and their derivatives, and phenylpropanoids and polyketides was higher in N7VF than in N7NF, while the content of the other compounds was higher in N7NF than in N7NF. These changes indicate that both leaves and fruits differ in their metabolic content. Furthermore, the organs of mutant and normal plants also differ in terms of metabolite content.

Principal component analysis showed that replicates of each sample were grouped together (Figure 2D). Similarly, hierarchical clustering showed that leaf samples from both N7N and N7V were clustered together, while fruit samples were clustered together (Figure 2E). The PCA further confirmed these observations, where replicates of the same tissue had a relatively higher correlation compared to the different tissues and plants (Figure 2F).

### 3.2 Comparative metabolome profile of N7VL and N7NL

**FIGURE 3:**
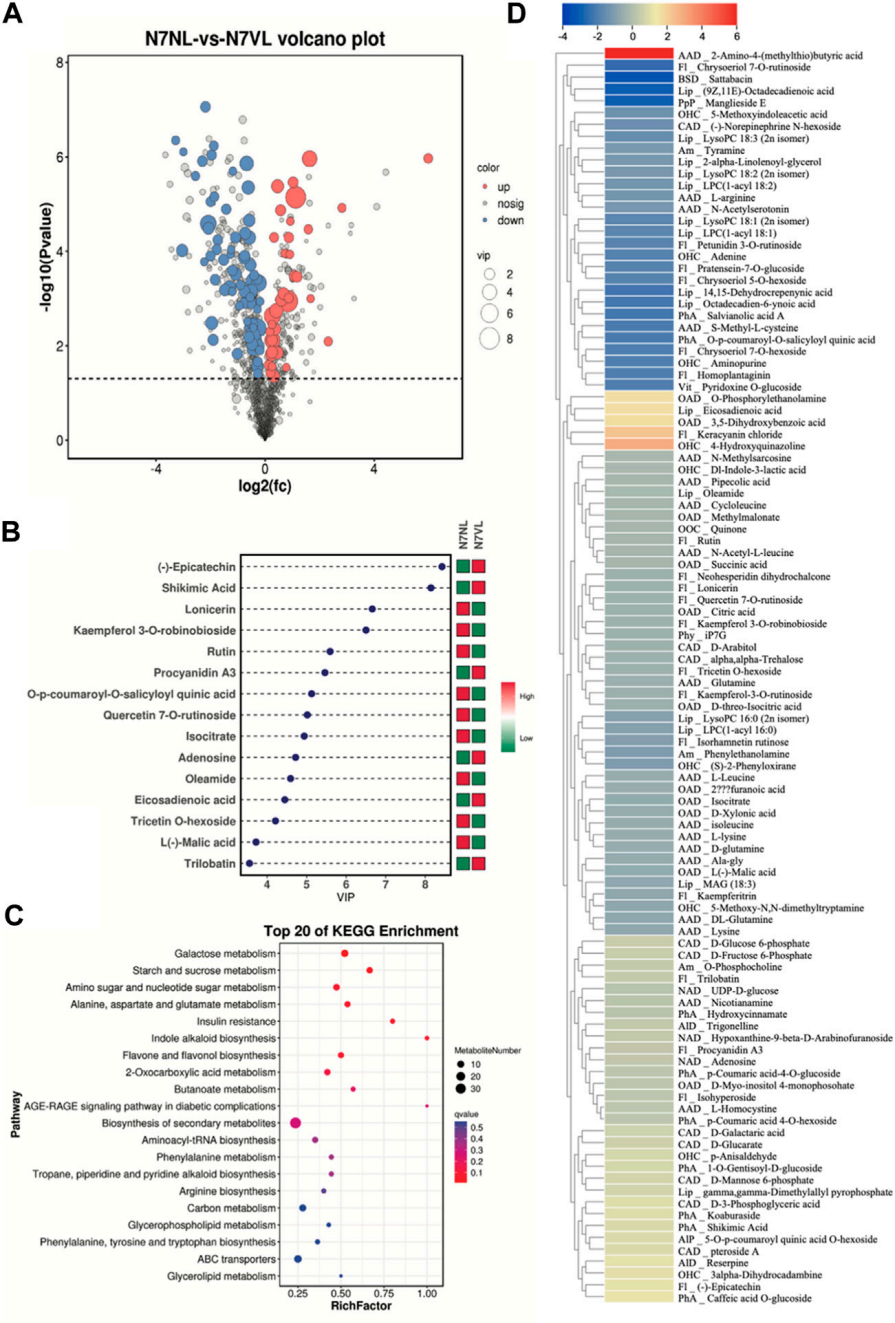
Comparative metabolome profiles of N7NL and N7VL. **(A)** Volcano plot of DAMs, **(B)** bar plot of the top 15 DAMs based on VIP scores, **(C)** scatter plot of KEGG pathways to which DAMs were enriched, and **(D)** heatmap of log2 fold change values of DAMs in N7NL vs. N7VL, where Alp denotes alcohols and polyols; AlD, alkaloids and derivatives; Am, amines; AAD, amino acids and derivatives; BSD, benzene and substituted derivatives; CAD, carbohydrates and derivatives; Fl, flavonoids; Lip, lipids; NAD, nucleotides and derivatives; OAD, organic acids and derivatives, OHC, organoheterocyclic compounds; OOC, organooxygen compounds; PhA, phenolic acids; Php, phenylpropanoids and polyketides; Phy, phytohormones; and Vit, vitamins.

The screening of metabolites based on VIP ≥ 1 and *t*-test *p* < 0.05 in the OPLS-DA model resulted in 106 DAMs between N7VL and N7NL, out of which 37 and 69 metabolites showed increased and decreased accumulation in N7VL compared to N7NL, respectively (Figure 3A). The top 15 metabolites based on the VIP of OPLS-DA are shown in Figure 3B. Notably, epicatechin, shikimic acid, procyanidin A3, adenosine, eicosadienoic acid, and trilobatin contents were higher in N7VL than in N7NL. The DAM with highest log2FC was 2-amino-4-(methylthio)butyric acid (5.99), followed by 4-hydroxyquinazoline (2.82), keracyanin chloride (2.31), 3,5-dihydroxybenzoic acid (1.67), and eicosadienoic acid (1.63).,while those having significantly higher contents in N7NL than in N7VL were sattabacin (−3.29), manglieside E (−3.05), (9Z,11E)-octadecadienoic acid (−3.0), chrysoeriol 7-O-rutinoside (−2.56), and 14,15-dehydrocrepenynic acid (−2.3). The DAMs in N7NL compared to N7VL were enriched in biosynthesis of secondary metabolites; starch and sucrose metabolism; citrate cycle; valine, leucine, and isoleucine degradation; glycerolipid metabolism; and others (Figure 3C).

Among the DAMs, most of the compounds classified as carbohydrates and derivatives, nucleotides and derivatives, and phenolic acids were accumulated in higher amounts in N7VL than in N7NL, where the higher carbohydrate content, i.e., D-fructose 6-phosphate, D-glucose 6-phosphate, D-glucarate, D-galactaric acid, D-mannose 6-phosphate, pteroside A, and D-3-phosphoglyceric acid in N7VL indicates higher resources for downstream processes. In addition, the higher levels of hydroxycinnamate, p-coumaric acid 4-O-glucoside, p-coumaric acid 4-O-hexoside, 1-O-gentisoyl-D-glucoside, shikimic acid, and caffeic acid O-glucoside in N7VL suggest that the mutant leaves are better in these traits. On the other hand, the content of most of the amino acids and derivatives, flavonoids, lipids and organic acids and derivatives, organoheterocyclic and organooxygen compounds, phenylpropanoids, phytohormone (N6-isopentenyladenine-7-glucoside, iP7G), and vitamins was lower in N7VL than in N7NL (Figure 3D). This change may be related to the higher cytokinin (iP7G) content (Wang et al., 2016).

### 3.3 Comparative metabolome profile of N7NF and N7VF

**FIGURE 4:**
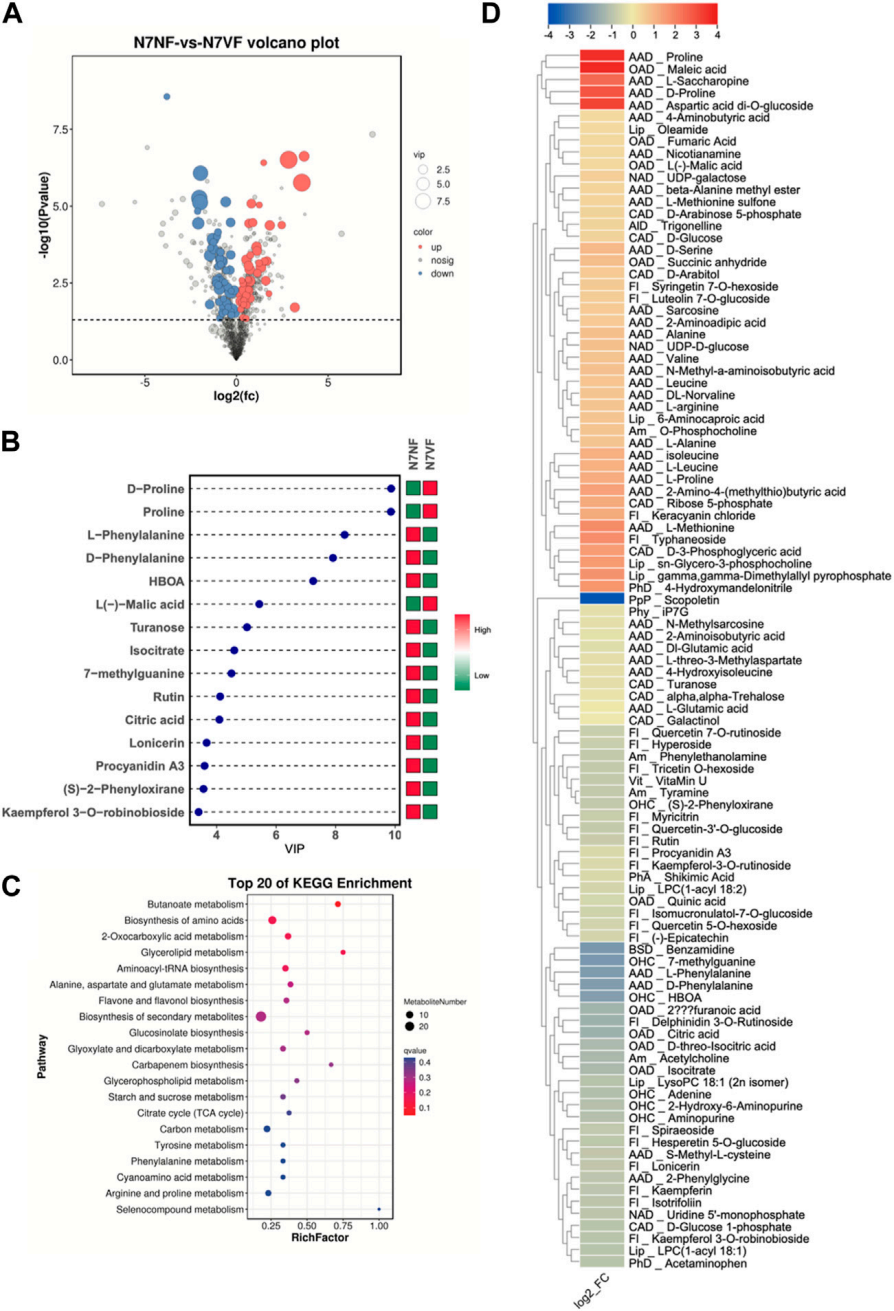
Comparative metabolome profiles of N7NF and N7VF. **(A)** Volcano plot of DAMs, **(B)** bar plot of the top 15 DAMs based on VIP scores, **(C)** scatter plot of KEGG pathways to which DAMs were enriched, and **(D)** heatmap of log2 fold change values of DAMs in N7NF vs. N7VF, where Alp denotes alcohols and polyols; AlD, alkaloids and derivatives; Am, amines; AAD, amino acids and derivatives; BSD, benzene and substituted derivatives; CAD, carbohydrates and derivatives; Fl, flavonoids; Lip, lipids; NAD, nucleotides and derivatives; OAD, organic acids and derivatives, OHC, organoheterocyclic compounds; OOC, organooxygen compounds; PhA, phenolic acids; Php, phenylpropanoids and polyketides; Phy, phytohormones; and Vit, vitamins.

The screening of metabolites yielded 101 DAMs, of which 45 and 56 showed increased and reduced accumulation in N7VF compared to N7NF, respectively (Figure 4A). The top 15 metabolites based on the VIP of OPLS-DA are shown in Figure 4B. N7VF had higher content of maleic acid (3.69), proline (3.56), aspartic acid (3.18), D-proline (2.84), L-saccharopine (2.47), L-methionine (1.82), typhaneoside (1.77), and others. On the contrary, N7NF had higher amounts of scopoletin, benzamidine, 7-methylguanine, L-phenylalanine, D-phenylalanine, HBOA, delphinidin-3-O-rutinoside, citric acid, and others. DAMs in N7NF compared to N7VF were enriched in butanoate metabolism, amino acid biosynthesis, 2-oxocarboxylic acid metabolism, glycerolipid metabolism, and others (Figure 4C).

N7VF fruits contained higher levels of amino acids and derivatives, carbohydrates and derivatives, and a limited number of compounds classified as lipids (oleamide, 6-aminocarboxylic acid, sn-glycero-3-phosphocholine, and gamma,gamma-dimethylallyl pyrophosphate) and organic acids and derivatives (L-malic acid, fumaric acid, succinic anhydride, and maleic acid). N7NF fruits contained higher levels of flavonoids, scopoletin, amines, some amino acids and derivatives (L-phenylalanine, D-phenylalanine, L-glutamic acid, 2-aminoisobutyric acid, etc.), benzamidine, carbohydrates and carbohydrate derivatives, (D-glucose-1-phosphate, turanose, alpha, alpha-trehalose, and galactinol), uridine 5′-monophosphate, some organic acids and derivatives (citric acid, furanoic acid, isocitrate, D-threo-isocitric acid, and quinic acid), organoheterocyclic compounds, shikimic acid, scopoletin, iP7G, and vitamin U (Figure 4D).

### 3.4 Transcriptome profiles of litchi leaf and fruit

**FIGURE 5:**
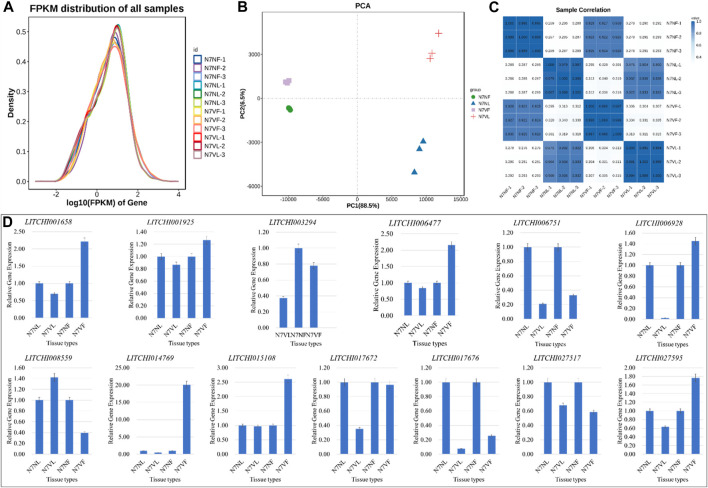
Transcriptome profile of litchi leaf and fruit. **(A)** Distribution of FPKM values, **(B)** principal component analysis, and **(C)** Pearson’s correlation coefficient of each replicate of leaf and fruit samples of the mother plant (N7N) and mutant (N7V). **(D)** Quantitative real-time PCR analyses of thirteen litchi genes in leaf and fruit samples of N7N and N7V, where L is leaf and F is fruit. The error bars on the graphs represent ± standard deviation (*n* = 3).

The sequencing of 12 libraries generated 513.84 million raw reads, with an average of 42.82 million raw reads per library. After filtering, 510.64 million clean reads (>99.1% of the raw reads) were obtained. On average, 81.56% of the clean reads could be mapped to the reference genome (Supplementary Table S1). In general, the FPKM distribution density was higher for N7NF and N7NV than for N7VF and N7VL (Figure 5A). The PCA showed a consistent grouping with that of the metabolomic samples, i.e., replicates were grouped according to samples. In particular, fruit samples showed less variation in PC2 compared to leaf samples. However, there was greater variability between the fruit and leaf (Figure 5B). This was also observed for PCA, i.e., there was a higher correlation between the expression in fruits of both samples compared to the fruit and leaf within a litchi species (Figure 5C). The qRT-PCR analysis results also complied with the RNA sequencing results (Figure 5D).

### 3.5 Differential gene expression in N7NL and N7VL

**FIGURE 6:**
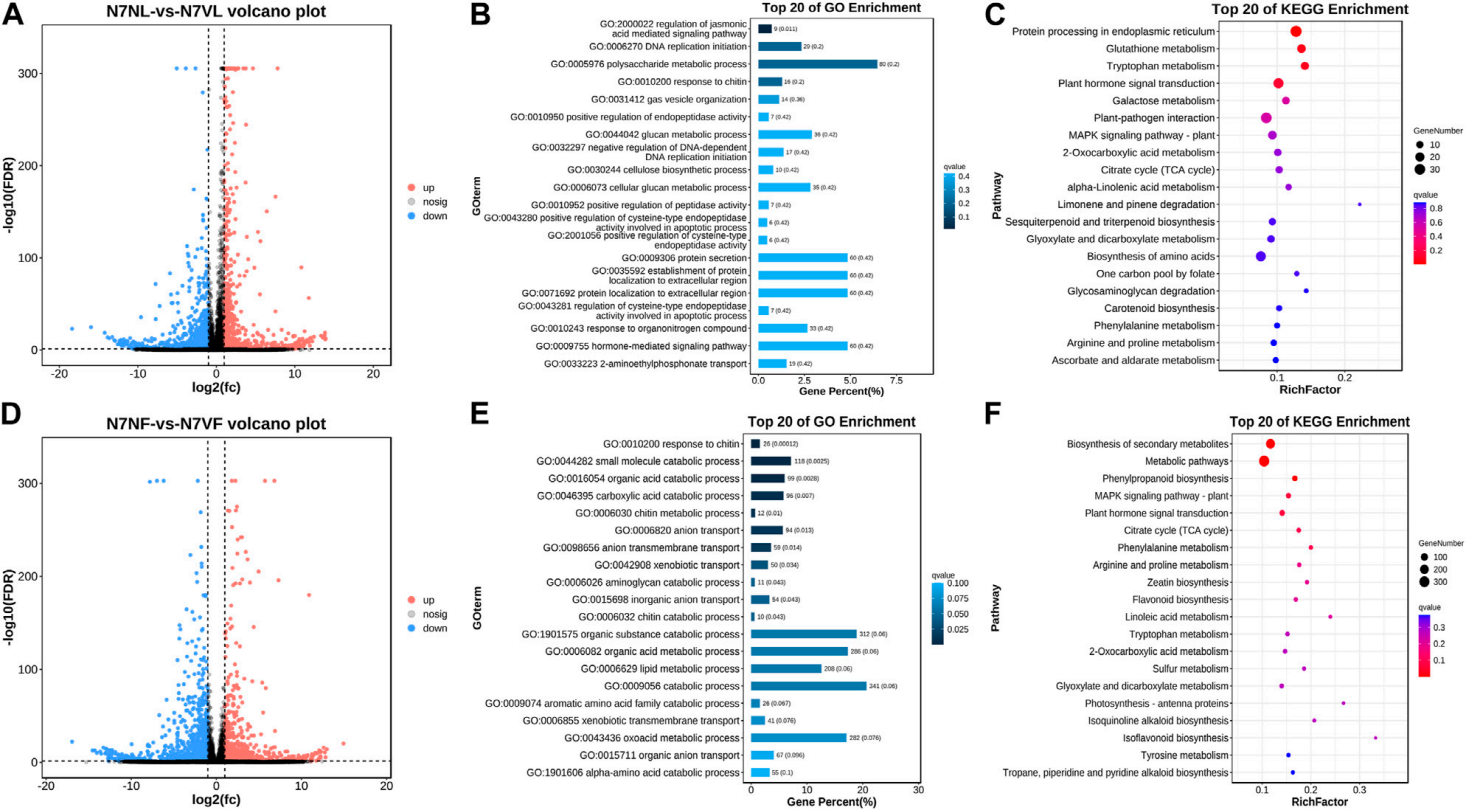
Differential gene expression in N7NL and N7VL. **(A)** Volcano plot of DEGs/DETs, **(B)** GO enrichment of DEGs/DETs, and **(C)** KEGG pathway enrichment of DEGs/DETs. **(D)** Volcano plot of DEGs/DETs, **(E)** GO enrichment of DEGs/DETs, and **(F)** KEGG pathway enrichment of DEGs/DETs.

Transcript screening revealed 2,144 DEGs/DETs, of which 895 and 1,249 had higher and lower expressions in N7VL than in N7NL, respectively (Figure 6A). The number of upregulated and downregulated genes is consistent with the observed number of DAMs. GO enrichment showed that the DEGs/DETs were mostly associated with biological processes (chitin response, small-molecule catabolism, organic acid catabolism, carboxylic acid catabolism, and chitin metabolism), molecular function (hydrolase activity, oxidoreductase activity, and tetrapyrrole binding), oxidoreductase activity, tetrapyrrole binding, heme binding, catalytic activity, chitin binding, aconite hydratase activity, 3-isopropylmalate dehydratase activity, and cellular component (cell periphery, plasma membrane, 3-isopropylmalate dehydratase complex, and the intrinsic component of the plasma membrane) (Supplementary Figure S1A). The DEGs/DETs were enriched in the GO process associated with polysaccharide metabolism processes, followed by hormone-mediated signaling pathways, protein secretion, protein localization, and glucan-mediated processes (Figure 6B). The DEGs/DETs were enriched in KEGG pathways associated with carbon metabolism; phenylpropanoid biosynthesis; biosynthesis of amino acids; terpenoid backbone biosynthesis; pentose and glucuronate interconversions; cutin, suberin, and wax biosynthesis; and carotenoid biosynthesis (Figure 6C). These observations are consistent with those of metabolome profiles of the two fruit types.

As the metabolite profiles indicated differences in carbohydrate-related compounds, we filtered the genes enriched in related pathways. Two genes enriched in the carbon fixation pathway in photosynthetic organisms, i.e., LITCHI027517, sedoheptulose bisphosphatase, and LITCHI014769, photosystem I subunit II, showed lower expressions in N7VL than in N7NL. In addition, 31 transcripts annotated as 21 genes enriched in carbohydrate biosynthesis-related pathways showed differential expression in the leaf samples of the two cultivars. In particular, we found that genes associated with the biosynthesis of D-glucose-6-P, D-fructose, trehalose, D-glucose, ADP-glucose, starch, and dextrin had higher expressions in N7VL than in N7NL. In the case of N7NL, transcripts associated with the biosynthesis of D-glucose, cellobiose-6-P, sucrose-6-P, dextrin, and maltose had higher expressions. Taken together, these observations indicate that both leaves had different expressions for carbohydrate biosynthesis-related genes, and the observed content of carbohydrate-related metabolites in N7VL may be related to these expressions (Supplementary Table S2).

In N7VL, we observed higher expressions of 5-O-(4-coumaroyl)-D-quinate 3′-monooxygenase (C3′H, LITCHI008559), consistent with higher shikimic acid and caffeic acid O-glucoside contents. In contrast, the increased accumulation of DAMs such as flavonoids and anthocyanins could be due to higher expressions of caffeoyl-CoA O-methyltransferase (LITCHI017676, LITCHI017674, and LITCHI017673) and isoflavone/4′-methoxyisoflavone 2′-hydroxylase (CYP81E, LITCHI006751) in N7NL. Similarly, consistent with the lower amino acid content in N7VL, we found that S-sulfo-L-cysteine synthase (LITCHI003294) and glycine hydroxymethyltransferase (glyA, LITCHI007175) had higher expressions in N7NL. However, several other transcripts associated with the GO term “vitamin” had higher expressions in N7VL, suggesting that more in-depth studies will be required to define the role of these genes and leaf-specific traits. Next, we searched for transcripts/genes enriched in amino acid-related pathways, as in general, amino acids and derivatives were highly accumulated in N7NL compared to N7VL. Consistent with the metabolomic observations, 19 out of 28 transcripts were highly expressed in N7NL compared to N7VL. In the case of transcripts enriched in lipid and related pathways, 7 out of 13 genes were highly expressed in N7NL compared to N7VL, confirming the metabolomic observations. Finally, two of the five genes associated with organic acid-related GO terms, i.e., PYR/PYL (LITCHI018118) and ATP-binding cassette, subfamily B (MDR/TAP), and member 1 (LITCHI026684), had higher expression in N7NL. In contrast, those highly expressed in N7VL were associated with a carbon pool through the folate pathway. These observations confirmed the metabolome-based results (Supplementary Table S2).

### 3.6 Differential gene expression in N7NF and N7VF

Transcript screening revealed 2,699 DEGs/DETs, out of which 1,547 and 1,152 had lower and higher expressions, respectively, in N7VF than in N7NL (Figure 6D). The number of upregulated and downregulated genes is consistent with the observed number of DAMs. GO enrichment showed that the DEGs/DETs were mostly associated with biological processes (cellular, metabolic, developmental, signaling, reproductive, growth, and pigmentation-related processes), molecular function (catalytic, binding, transporter, transcriptional regulation, ATP-dependent, antioxidant, and nutrient reservoir activities), and cellular component (cellular anatomical unit and protein-containing complex) (Figure 6E; Supplementary Figure S1B). DEGs/DETs were enriched in secondary metabolite, phenylpropanoid and flavonoid biosynthesis, and the phytohormone and MAPK signaling pathway (Figure 6F).

As fruit development is influenced by hormone levels and signaling, we searched for DEGs/DETs enriched in related pathways. Eleven out of 37 transcripts enriched in plant hormone signaling and plant MAPK pathways had higher expressions in N7VF than in N7NF. These transcripts were related to auxin (AUX1, AUX/IAA, and GH3), cytokinin (CRE1), GA (PIF3/PIF4), ABA (PYR/PYL), and JA (MYC2) signaling. Interestingly, those with higher expressions in N7NF than in N7VF were also associated with auxin (TIR1, AUX/IAA, ARF, and GH3), cytokinin (B-ARR), GA (DELLA), ABA (PYR/PYL), ethylene (ETR and ERF1/2), BR (BIN2 and TCH4), JA (MYC2), and SA (PR-1) signaling. These observations suggest that hormones may play a role in the differences in leaf and fruit phenotypes (Supplementary Table S2). Considering the observation that N7VF had a higher total carbohydrate content, we searched for genes associated with pathways related to carbohydrate biosynthesis. The transcripts were annotated as trehalose-6-phosphate synthase (ostA, *LITCHI020469*), glucose-1-phosphate adenylyltransferase (glgC, *LITCHI014739*), endoglucanase (EG, *LITCHI001925* and *LITCHI022015*), beta-glucosidase (BGLU, *LITCHI024092*), and glucan endo-1, 3-beta-D-glucosidase (EGLC, *LITCHI017822*). In contrast, those involved in the biosynthesis of D-glucose-6P, D-fructose, trehalose, D-glucose, and cellobiose had higher expression in N7NF, indicating that a complex network of genes is involved in the differential carbohydrate biosynthesis in the two fruit types. There were also DEGs/DETs enriched in galactose metabolism, fructose and mannose metabolism, gluconeogenesis, galactose metabolism, and pentose and glucuronate interconversion. Thus, the observed differences in carbohydrate content between the two fruits also involve these pathways.

Twenty-four out of the 45 DETs enriched in pathways related to amino acid biosynthesis had higher expressions in N7NF than in N7VF. The others had higher expressions in N7VF than in N7NF. These pathways include metabolism pathways such as glutathione metabolism, cyanoamino acid metabolism, amino acid biosynthesis, valine, leucine, and isoleucine biosynthesis, and phenylalanine, tyrosine, and tryptophan metabolism. The higher content of lipid-related metabolites is consistent with the higher expression of 12 out of 21 DETs, which are enriched in glycerolipid metabolism, ether lipid metabolism, and glycerophospholipid metabolism. As most organic acid-related metabolites had higher levels in N7NF than in N7VF, we observed consistent gene expression. Sixty-three of the 117 DEGs/DETs associated with the GO term “organic acid-related functions,” showed higher expressions in N7NF than in N7VF (Supplementary Table S2).

Since N7NF had a higher flavonoid content, we looked for DETs enriched in related pathways. In particular, caffeoyl-CoA O-methyltransferase (CCOMT, LITCHI017676, and LITCHI001586) and C3′H (LITCHI008559) had higher expression in N7NF. On the other hand, N7VF had higher levels of several compounds such as flavonoids including luteolin 7-O-glucoside, syringetin 7-O-hexoside, keracyanin chloride, and typhaneoside. These higher levels may be due to the relatively higher expression of chalcone isomerase (CHI, LITCHI027595), chalcone synthase (CHS, LITCHI020852 and LITCHI015108), CCOMT (LITCHI017672), anthocyanin reductase (ANR, LITCHI029356), naringenin 3-dioxygenase (F3H, LITCHI006477), and others. Overall, the transcriptome data are consistent with the metabolome profiles of the two fruits (Supplementary Table S2).

## 4 Discussion

Litchi is one of the most important tropical fruits in the world. It has been cultivated in China for more than 2,000 years and gained an important status. Particularly, it is cultivated in Guangdong, Guangxi, Fujian, and Sichuan provinces of China (Sun et al., 2021). Some of the major fruit-related breeding traits are fruit shape, size, taste, color, and perishability (Sun et al., 2010). During the ongoing litchi breeding program, we observed a mutant originated from budding which had differences in fruit and leaf shape/appearance. The fruit peel cracking of the mutant is smooth, and it matures 7–10 days earlier than N7N. Such a peel is relatively conducive to storage conditions for the litchi fruit. The better taste and early maturity are promising traits to capture the market. Currently, there are two types of turtle split fruit on the litchi market. Among them, Guiwei is the representative variety of the fruits with peak-type peel cracks, and Nuomici is the representative variety of the fruits with smooth peel cracks. These two varieties are the most popular in the market. The characteristic of the peak protrusion of the fissure film has no effect on consumers. In the case of leaves, the mother plant had twisted leaves, but the mutant showed smooth and flattened leaves (Figure 1). The folded leaf phenotype in N7NL could be responsible for lower photosynthetic potential due to light capture compared to the smooth leaf phenotype. In this regard, the foremost task is to understand whether these two organs contain useful differences in metabolome and respective transcriptome or not. This information is important to decide whether to include it in the breeding program and explore its traits in detail or not.

The detection of the same metabolites in both the mother plant and mutant clearly indicates that they are genetically similar, where the higher quantities of several metabolites such as carbohydrates and derivatives (D-fructose 6-phosphate, D-glucose 6-phosphate, D-glucarate, D-galactaric acid, D-mannose 6-phosphate, pteroside A, and D-3-phosphoglyceric acid), nucleotides and derivatives, and phenolic acids in N7VL suggest that the mutant could possibly have better potential for carbon assimilation and energy production. The higher carbohydrate content is not only linked with improved flower induction (Corbesier et al., 1998) but also has been associated with sink nutrition (Wibbe and Blanke, 1995). The transcriptome sequencing suggested that these changes are possibly due to differential expressions of carbohydrate biosynthesis-related genes (those enriched in starch and sucrose metabolism) (Hirose et al., 1999). However, the lack of data on leaf curvature and its causes makes comparisons difficult. In addition, there are several other factors that can influence leaf curvature in addition to genotype differences (Briglia et al., 2020; Chen et al., 2023). Nevertheless, our results provide preliminary data on differential metabolome and transcriptome profiles. These genes are prime targets for functional characterization. In the case of the mother plant, the presence of higher quantities of amino acids and derivatives, flavonoids, lipids, organic acids, and iP7G indicates that N7NL mutants have several beneficial metabolites. Flavonoid accumulation has been proven useful again several biotic and abiotic stress factors in multiple tree species, e.g., they increase the tolerance of apple leaves against rust (Lu et al., 2017), pathogen resistance in poplar (Bai et al., 2020), and drought resistance in sea buckthorn (Gao et al., 2021) and 11 other tree species (Bhusal et al., 2021). Our observation that N7NL mutants have higher flavonoid content is consistent with the expressions of caffeoyl-CoA O-methyltransferase (LITCHI017676, LITCHI017674, and LITCHI017673) and CYP81E. These observations are also consistent with the described roles of caffeoyl-CoA O-methyltransferase (Liu et al., 2020) and CYP81E (Liu et al., 2003) in flavonoid and isoflavone biosynthesis. Therefore, these genes can be further characterized for exploration of their role in litchi leaf flavonoid contents.

Similarly, the higher contents of other metabolites like amino acids and organic acids in leaves lead to better tolerance against stresses (Šircelj et al., 2005). The higher organic acid content is possibly due to the higher expression of PYR/PYL (LITCHI018118), which plays a role in changing the organic acid profile by abscisic acid biosynthesis (Khosravi-Nejad et al., 2022). Moreover, the higher expression of MDR/TAP member 1 in N7NL can also be a possible explanation for relatively higher organic acid biosynthesis. However, this should be further characterized in both types of leaves, and its specific role should be explored. The higher contents of Ip7G and regulation of several phytohormone signaling sub-pathways, i.e., auxin, GA, JA, SA, BR, and ETH, are consistent with the accumulation of flavonoids, carbohydrates, and other secondary metabolites in the mother plant. Earlier studies have clearly indicated that cytokinins (and/or auxins) have flavonoid-dependent modulation in plant growth (Kurepa et al., 2023). Similarly, carbohydrates, flavonoids, anthocyanins, phenols, and chlorophyll contents are affected by cytokinin application (Hossain et al., 2023). From these observations, it can be concluded that the differences in the mother plant and mutant metabolome are potentially related to phytohormones. Overall, the two leaves contain different levels of primary and secondary metabolites, which are associated with the differences in the expression of genes enriched in the respective pathways.

Earlier works on litchi metabolomics have shown that amino acid content in pulp is related to the storage of litchi; therefore, our results that the bioactive compounds between the mother plant and mutant fruits have different accumulation trends are useful data (Guo et al., 2019). The observations that N7VF contained a higher content of compounds including maleic acid, proline, aspartic acid, several other amino acids, and carbohydrates are highly valuable in terms of fruit taste and health benefits (Zhao et al., 2020; Sun et al., 2021). Litchi fruits that have higher soluble sugar content are sweeter in taste. Earlier work on litchi fruit using multi-omics techniques revealed that DEGs enriched in several carbohydrate metabolism-related pathways are involved in the biosynthesis of carbohydrates and derivatives (Guo et al., 2022). Here, the higher expression of ostA (in N7NF) responsible for trehalose-6-phosphate biosynthesis is consistent with the increase in carbohydrate biosynthesis. Trehalose-6-phosphate plays a central role in carbohydrate biosynthesis regulation (Ponnu et al., 2011), whereas glgC is involved in ADP–glucose biosynthesis (GO:0008878). Thus, the higher total carbohydrate content in N7VF could be a result of expression differences of ostA, glgC, EG, BGLU, EGLC, and of several other genes discussed. Moreover, long-term storage of litchi affects the organic acid, amino acid, and carbohydrate contents in fruit pulp (Guo et al., 2023). Therefore, the noticeable differences in accumulation of these compounds in N6NF and N7VF lay the foundation for further studies. Finally, the observations that several genes are enriched in hormone biosynthesis as well as signaling pathways indicate the potential roles of auxin, cytokinin, GA, ABA, JA, BR, and ethylene in the differences in fruit phenotypes. Phytohormones are integral to fruit development and maturation processes; therefore, these datasets highlight the need for functional characterization of individual genes with exogenous application of individual phytohormones (Fenn and Giovannoni, 2021). Moreover, the phytohormone content as well as the fruit quality can also be influenced by environmental factors and agronomic practices (do Nascimento Nunes, 2008; Kumar, 2012); therefore, these preliminary datasets should also be further validated in specific environmental conditions. Moreover, the fruit cracking could also be caused by the differential regulation of phytohormones. An earlier study had indicated that changes in hormone balance can constitute the molecular basis of fruit cracking susceptibility in litchi fruits (Wang et al., 2019). Apart from these, fruit cracking in litchi has also been associated with pericarp photosynthesis and the oxidation of unsaturated fatty acids (Wang et al., 2019). Overall, our combined transcriptome and metabolome analysis indicates that the two fruit types have different flavonoid, amino acid, carbohydrate, and organic acid contents owing to differences in the expression of related genes. These observations lay the foundation for future work on understanding individual traits, i.e., taste, appearance, shelf life, and fruit color.

## 5 Conclusion

During our ongoing litchi breeding program, a mutant (as a result of budding) was produced. The mutant had flat leaves, whereas the mother plant had curved leaves. The fruits’ peel had prominent cracks compared to the mother plant. Based on the combined omics analysis, we conclude that the leaves and fruits of the mother plant and mutant differ in the metabolome profiles. We conclude that the mutant fruits are richer in carbohydrates and derivatives and amino acids and derivatives, whereas the fruits from mother plant contain a higher content of flavonoid, phenolic acids, scopoletin, amines, some amino acids and derivatives, benzamidine, carbohydrates and carbohydrate derivatives, organic acids and derivatives, shikimic acid, scopoletin, iP7G, and vitamin U. The transcriptome profile indicates the possible roles of genes enriched in several pathways associated with carbohydrate biosynthesis/metabolism, amino acid metabolism, flavonoid biosynthesis, phenylpropanoid biosynthesis, plant hormone, and MAPK signaling pathways.

## Data Availability

The original contributions presented in the study are publicly available. These data can be found at: https://ngdc.cncb.ac.cn/bioproject/browse/PRJCA022111.

## Supplementary material

SUPPLEMENTARY FIGURE S1 Classification of GO terms (biological process, cellular component, and molecular function) associated with differentially expressed genes in **(A)** N7NL vs. N7VL and **(B)** N7NF vs. N7VF.
