# Supplementary figures and images for: Comparative transcriptome and metabolome profiles of the leaf and fruits of a Xianjinfeng litchi budding mutant and its mother plant
Source: Front Genet. 2024 Feb 23;15:1360138. doi: 10.3389/fgene.2024.1360138 (PMC10920226; doi:10.3389/fgene.2024.1360138)

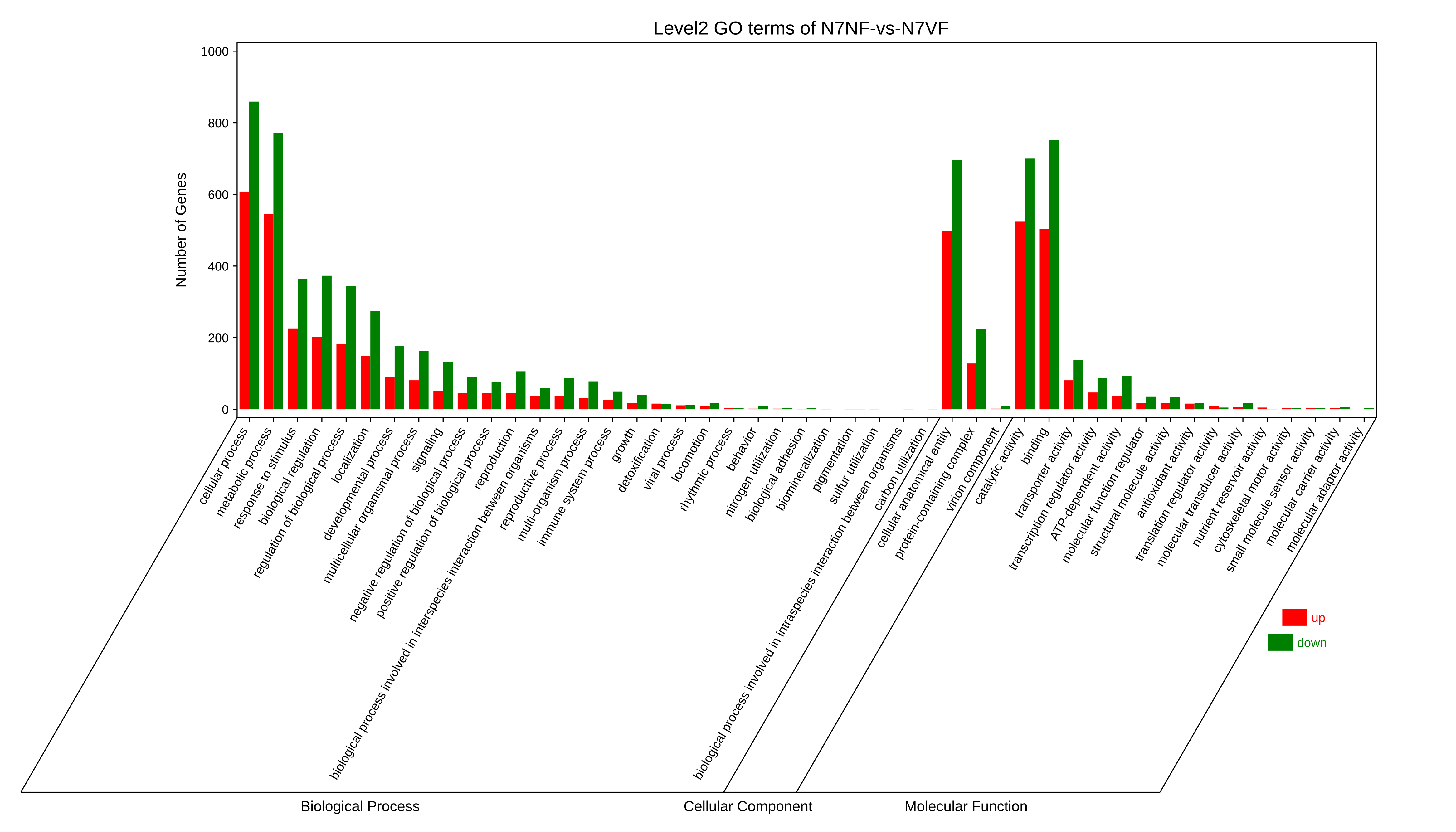

Supplement: Supplementary file 1 [file Image2.PNG]

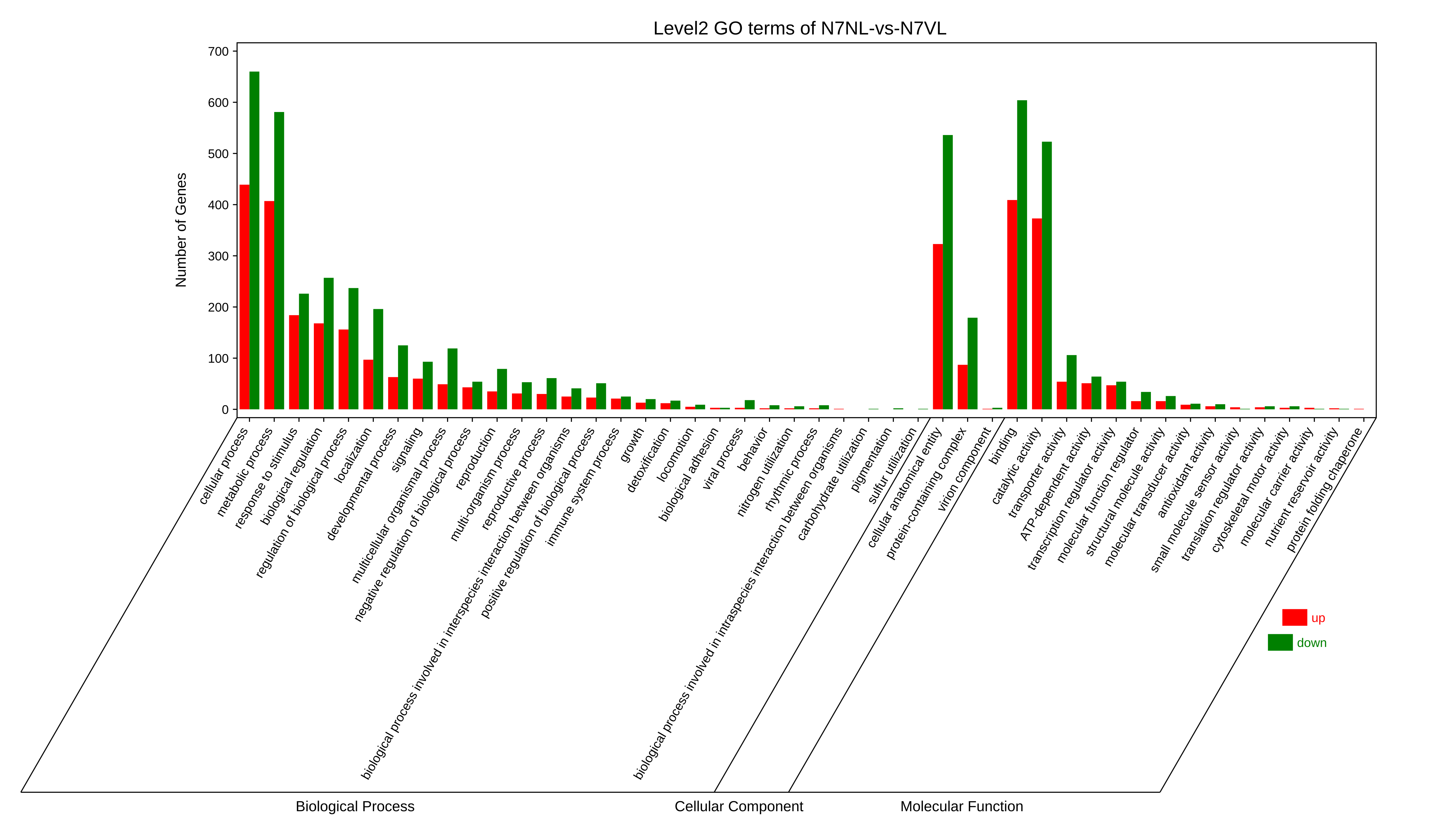

Supplement: Supplementary file 3 [file Image1.PNG]
